# Digital Competence among Healthcare Leaders: A Mixed-Methods Systematic Review

**DOI:** 10.1155/2024/8435248

**Published:** 2024-07-30

**Authors:** Noora Laakkonen, Erika Jarva, Mira Hammarén, Outi Kanste, Maria Kääriäinen, Anne Oikarinen, Kristina Mikkonen

**Affiliations:** ^1^ Research Unit of Health Sciences and Technology Faculty of Medicine University of Oulu, Oulu, Finland; ^2^ The Finnish Centre for Evidence-Based Health Care: A Joanna Briggs Institute Centre of Excellence, Oulu, Finland; ^3^ Medical Research Center Oulu University Hospital, Oulu, Finland

## Abstract

**Background:**

New evidence on the digital competencies of healthcare leaders can provide essential knowledge for building training for the leaders to ensure high-quality patient care.

**Objective:**

The aim of this mixed-methods systematic review was to identify the current best evidence from qualitative, quantitative, and mixed-methods studies on healthcare leaders' digital competence experiences and perceptions and factors associated with it.

**Methods:**

A mixed-methods systematic review was conducted following the Joanna Briggs Institute guidelines for mixed-methods systematic reviews by including original qualitative and quantitative observational studies and mixed-methods studies published in English or Finnish between January 2012 and January 2024. The studies were retrieved from four databases (CINAHL, PubMed, Scopus, and Medic). In total, 4470 articles were screened, 122 were eligible for full-text screening, and 19 articles were included in the review according to the established inclusion and exclusion criteria. *Data Extraction and Synthesis*. Data tabulation and narrative synthesis for quantitative studies and content analysis for qualitative studies.

**Results:**

The synthesis of qualitative data identified five main categories that describe healthcare leaders' experiences with digital competencies: (1) the need for developing leader's own, professionals', and patients' competence in the digitalisation of healthcare, (2) the need for expertise in the health IT implementation process, (3) positive perceptions towards technology, (4) negative perceptions towards technology, and (5) ability to act as an advocate to implement technology into practice. Data from the selected quantitative studies presents that factors associated with the digital competence of healthcare leaders include individual characteristics, career characteristics, training, and other factors.

**Conclusion:**

This review suggests that developing and supporting healthcare leaders' digital competencies should be considered in healthcare organizations, research, and education to make their digital competencies meet the demands of increasingly digitalising healthcare development work.

## 1. Introduction

Digitalising services and their integration into patient care challenge the competence needed in social and healthcare [1]. The lack of education and training of healthcare leaders may explain the low success rate of implementing digital solutions in healthcare. Previous research has found that managers are often self-taught in the use of digital health services [2], and a knowledge gap has been recognised in the healthcare leaders' digital competencies in the rapidly evolving and digitalising healthcare environments [3]. The digital working culture of healthcare has changed rapidly due to the coronavirus pandemic, for example, in digital work interaction. Primary healthcare leaders predict that digitalisation in healthcare will develop further [4]. Digitalising healthcare requires adaptation to the requirements and needs of the changing working life, both from employees and leaders [5], and to fully participate in healthcare management in an era of rapidly evolving health information technologies, nursing leaders need to acquire health information technology skills related to their clinical leadership roles and responsibilities [3, 6]. The European Commission [7] defines digital competence as “the confident and critical use of Information Society Technology (IST) for work, leisure and communication…underpinned by basic skills in information technology to retrieve, assess, store, produce, present and exchange information, to communicate and participate in collaborative networks via the Internet.” Mainz et al. [8] reported in their review that digital competence in healthcare can be divided into technical, methodological, personal, and social skills. The technical competencies identified encompass proficiency in basic computer use and wireless devices, applied skills in digital health, anticipation of advanced digital capabilities, technology administration, ethical considerations, and legal aspects of digitalisation. Methodological competencies include proficiency in data and information processing, problem-solving abilities, continuous learning, project management, and research skills. Self-reflection, critical thinking, professionalism, creativity, and innovative behaviour are considered to be personal competencies; and focus on patients, working in teams, communication competence, teaching, and networking skills are categorised as social competencies in digital competence in healthcare.

Health information technologies have good potential to improve the quality, safety, patient-centeredness, and cost-effectiveness of treatment [9]. In the increasingly digitalising healthcare environments, the need for competence management of nursing leaders has been emphasised as healthcare organizations acquire and implement different technologies and health information systems to support nursing work. Nursing leaders need to participate in decision-making in the selection, planning, deployment, and evaluation of these technological procurements (Reference [10]). Due to the rapid integration of digital health technologies, healthcare leaders must have the expertise and confidence to implement and incorporate these technologies into healthcare [11] to support healthcare professionals' digital competence and thus contribute to ensuring quality patient care [12]. According to Carson et al. [13], nursing leadership is crucial in enhancing electronic health record (EHR) satisfaction and usability by fostering a positive culture of staff engagement and promoting EHR adoption. Proficiency in EHRs is essential for nursing leaders to serve as role models for staff, enabling effective communication and support for evolving functionalities that enhance patient care workflows [13]. Due to some nurses' insufficient digital competence, hospital management and nurse leadership need to recognise the critical role of aligning technology with tasks and individuals to ensure the successful adoption of health information technology [14]. Healthcare leaders and professionals have highlighted the importance of leaders' role and digital competence in enhancing and disseminating digital skills; leaders need to grasp the principles and functionalities of digital tools and devices and understand their potential benefits for professionals and patients [15, 16].

Previous research has shown that healthcare leaders need help and training in using and introducing technology in their work community [2, 17, 18]. It has been found that healthcare leaders have not always received training in information technology, or training has been insufficient [6, 19], for example, in the use of ICT and digital healthcare applications and programs [2]. According to Ravelin et al. [4], attention must be paid to the digital competencies of healthcare leaders because, according to their experience, leaders must first have capability in the use of information technology so that they can guide employees in the use of information technology. Healthcare leaders need capabilities and contextual competencies to effectively utilise complex digital tools in a changing environment, alongside improving digital health literacy, to inform strategic and operational decisions [20]. However, healthcare leaders continue encountering various barriers, such as infrastructure, technical, training, legal, ethical, time, and workload issues, when using digital health technologies, irrespective of care level or specific technology [21].

There is widespread optimism regarding artificial intelligence's (AI) potential to enhance healthcare across diagnostics and treatment [22, 23]. When applied to nursing practice, AI can be a practical resource for leaders yielding positive outcomes in patient care and safety [24]. Also, Laukka et al. [25] suggest that AI can induce benefits on various areas of health care, including the work of nursing leaders. AI is ready to support healthcare workers across administrative tasks, clinical documentation, patient outreach, and specialised areas like image analysis and medical device automation [22]. AI systems offer notable potential in supporting comprehensive health services management and can aid doctors, nurses, and leaders in their roles [26]. For example, the integration of AI-driven triage systems can optimise operations in primary care and thus induce more proactive and tailored approach to healthcare delivery [23]. According to Neher et al. [27], healthcare leaders gearing up for AI implementation recognised its potential to improve healthcare, holding high expectations for its relative advantage; however, they were less certain about its evidence base, particularly regarding safety and effectiveness. Additionally, leaders were cautious about factors like trialability, adaptability, design, and costs [27]. Moreover, leaders have been reported to be concerned with the organization's internal capacity for strategic change management and the possibilities to amplify competence and expertise in the use of AI systems [23]. Successful AI implementation and integration require a team with diverse expertise on AI, collaboration within and beyond the healthcare realm, and the development of an organisation-wide AI innovation culture where healthcare leaders play a significant role [23, 28].

In recent years, there has been more focus put on the digital competence of healthcare professionals at large [8, 12, 29–33], but in most cases, healthcare leaders have not been the main subject of the study, or their results have not been separated from other healthcare professionals' results which makes it difficult to interpret specifically leaders' digital competence areas or needs. Certain studies and reviews [3, 6, 34] have specifically looked at the digital competence of healthcare leaders, focusing on knowledge and skills, dismissing other aspects related to digital competence, such as personal and social skills. Examining these different aspects of healthcare leaders' digital competence is essential as it influences employees' acceptance and usage of technology [35, 36], which has positive outcomes on patient treatment and processes [31]. To enable innovative service provision in digital healthcare, there is a need to enhance the digital competence and understanding of digital transformation among current and future healthcare leaders [11]. Synthesis of the evidence on the digital competence of healthcare leaders can provide essential knowledge for building further training for the leaders and their employees to ensure high-quality patient care in digitalised healthcare environments. For this reason, this mixed-method systematic review aimed to gather the most recent evidence on healthcare leaders' digital competence perceptions and associated factors.

## 2. Materials and Methods

### 2.1. Study Design

The Joanna Briggs Institute guidelines for mixed-methods systematic reviews [37] were followed in the implementation of this review. This mixed-methods systematic review protocol is registered with the Center for Open Science incorporation OSF (Open Science Framework) registries [38]. In this study, healthcare leaders refer to all persons positioned as managers or leaders in social and healthcare contexts, such as nurse leaders, nurse managers, and chief nursing officers. A mixed-method review was conducted to explore qualitative evidence on healthcare leaders' experiences relating to digital competence and synthesise quantitative findings from previous research concerning digital competence areas and factors associated with leaders' digital competence.

The research questions were

1. What kind of experiences do healthcare leaders have with digital competence?
2. What kind of digital competencies have been described for healthcare leaders?
3. What kind of factors are associated with the digital competencies of healthcare leaders?

### 2.2. Search Strategy

Original qualitative and quantitative observational studies and mixed studies, including both methodologies published in English or Finnish between 2012 and 2022, were included in this review. The year limitation was set because of rapid digitalisation development in healthcare since the last decade [39]. Yet, a stricter timeframe was neglected as the phenomenon of knowledge, skills, and attitudes related to the use of IST has a significant history. The studies were initially retrieved from four databases (CINAHL, PubMed, Scopus, and Medic) in October 2022. An additional search was conducted at the beginning of January 2024 to include the most recently published publications. Data search strategies, including used keywords and complete search phrases for used databases, are presented in supplementary file 1. Inclusion and exclusion criteria were defined before performing a database search according to PICo [37] and PEO [40] criteria. An information specialist was also utilised during this stage of the research. Inclusion criteria were (P) participants in different roles of healthcare leaders and managers at all levels of the organization; (I) & (O) the phenomenon of interest included digital competence (knowledge, skills, and attitudes related to the use of IST); and (Co) the context of healthcare organizations. For quantitative studies, (E) exposure of interest included factors related to the digital competence of healthcare leaders at all levels of the organization. Exclusion criteria included participants other than leaders or managers, e.g., social and healthcare professionals, for phenomena of interest or outcomes other than digital competence, and for contexts other than healthcare organizations. Study protocols, reviews, conference papers, books, and nonscientific research articles and texts published before 2012 in languages other than English or Finnish were also excluded.

### 2.3. Search Outcomes

**Figure 1:**
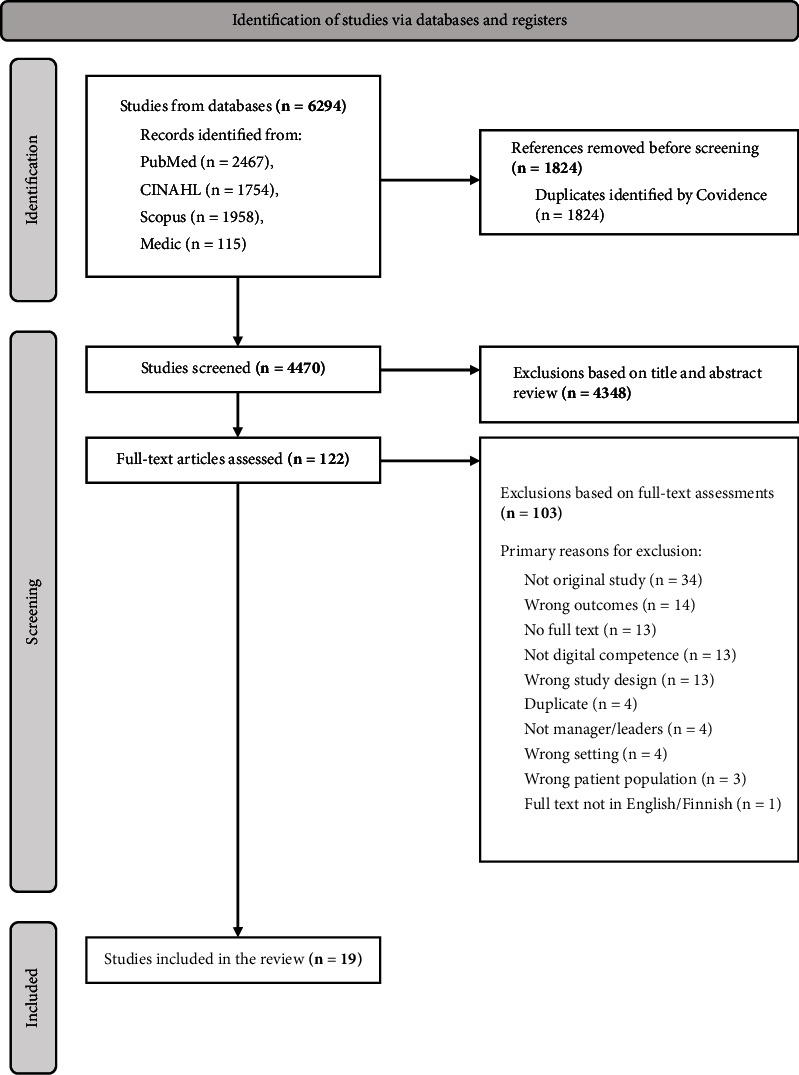
PRISMA flow diagram (Center for Reviews and Dissemination (CRD), 2009).

The database search resulted in in all 6294 studies. Among these studies, 1824 duplicates were found and removed. In total, 4470 studies were screened by title and abstract, and 122 were eligible for full-text screening. In addition, the references of chosen studies selected for the review were checked against the inclusion and exclusion criteria, but this did not add any more studies to this review. Each researcher conducted the screening process independently, and conflicts were solved via discussion or by a third party until agreement was reached. A total of 19 studies were agreed to be included in the mixed-methods systematic review, including seven cross-sectional, ten qualitative, and two mixed-methods studies (Figure 1) against the inclusion criteria [41].

### 2.4. Quality Appraisal

The methodological quality of the selected 19 studies relevant to the inclusion criteria was assessed using The Joanna Briggs Institute (JBI) Critical Appraisal tools; a checklist for analytical cross-sectional studies [40] including eight assessment criteria was used for quantitative studies (*n* = 7), and a checklist for qualitative research [42] including ten assessment criteria was used for qualitative studies (*n* = 10). Mixed-methods studies (*n* = 2) were assessed using both of these checklists. Four researchers (blinded for review) assessed the methodological quality of the studies separately and discussed possible disagreements in assessments until an agreement was reached. Quality assessment (maximum points 100%) for quantitative studies resulted in a score of 75% in four studies [43–46], 62.5% in one study [47], and 50% in two studies [48, 49]. For qualitative studies, a score of 100% was attained by one study [50], seven studies attained 80% [2, 51–56], and 70% by two studies [57]; Simpson., 2013. The mixed-methods studies by Kujala et al. [58] scored 75% for the cross-sectional and 60% for the qualitative approach, and Mottelson et al. [59] scored 37.5% for the cross-sectional and 80% for the qualitative approach. The detailed results from the methodological quality appraisal are presented in supplementary file 2.

### 2.5. Data Extraction and Synthesis

Data extraction, including authors, the year of publication, the country of origin, purpose, participants, methodology, phenomena of interest or exposure of interest, outcomes, and the key findings, was assessed from all the articles included in the review (Table 1). The selected quantitative studies were synthesised using data tabulation and narrative synthesis. Qualitative studies were analyzed with content analysis to synthesise the results from healthcare leaders' digital competence experiences and descriptions [61].

## 3. Results

### 3.1. Characteristics of the Studies

The original studies were conducted in the USA (*n* = 5), Finland (*n* = 3), Portugal (*n* = 2), Norway and Finland (*n* = 1), Indonesia (*n* = 1), Portugal and Brazil (*n* = 1), Ghana (*n* = 1), Qatar (*n* = 1), Turkey (*n* = 1), Japan (*n* = 1), China (*n* = 1), Denmark (*n* = 1), and Australia (*n* = 1). The study settings included specialised or university affiliated hospitals (*n* = 6), public or general hospitals and healthcare organizations (*n* = 5), a mixture of specialist, primary and private hospitals and health centers providing health and social care services (*n* = 4), private hospitals (*n* = 2), and a nursing home (*n* = 1). Two studies [56, 60] included participants from national health management networks. The studies included a total of 1639 participants who were healthcare leaders in different positions and levels, such as nursing unit managers, executive directors, assistant executive directors, directors of nursing, head nurses, nurse managers, nurse leaders, charge nurses in managerial positions, service managers, frontline leaders, and chief nurse executives.

### 3.2. Healthcare Leaders' Experiences with Digital Competence Based on Qualitative Data Synthesis

The selected qualitative studies examined the following areas of digital competence: (1) the need for developing the leader's own, professionals' and patients' competence in the digitalisation of healthcare, (2) the leader's need for digital competence and expertise in the health IT implementation process, (3) positive perceptions towards technology, (4) negative perceptions towards technology, and (5) ability to act as an advocate to implement technology into practice (Table 2).

#### 3.2.1. The Need for Developing Leader's Own, Professionals', and Patients' Competence in the Digitalisation of Healthcare

The first main category entailed the categories of knowledge and information needs in eHealth and health IT and the need for knowledge of the evidence base and regulatory expertise. In the study by Kujala et al. [58], leaders expressed that they cannot support their employees and patients without knowing about the new eHealth services. Leaders brought out their need to gain information and knowledge about eHealth services and their benefits and possibilities. Functionality, interoperability, usability of the eHealth services, and shortage of equipment and time resources generally in eHealth were considered challenging. According to Bezboruah et al. [57], leaders need to gain knowledge of health IT types and forms available, clinical and administrative health IT and their maintenance and potential exploitation in the facility, and health IT's challenges and benefits. They had concerns about customising the software functionality and choosing the right vendors for health IT. Moreover, leaders saw participating in technology development as one core competency area [50].

Leaders lacked knowledge about data availability and perceived there was a lack of appropriate user education and guidance in the use of data [56]. Leaders in the Bezboruah et al. [57] study lacked information regarding health IT software's usefulness, utility before, and ease of use after adoption. Insufficient information on health IT systems and their usage resulted in inadequate communication with the personnel and challenges in mutual understanding, which hindered health IT adoption. Gunawan et al. [50] report that leaders perceived technological skills' improvement as necessary. Leaders brought out the need to understand technology and how to use technological equipment, the knowledge of how to use technological tools to support their work, and the need to understand online systems to guide patients in their use. They also expressed the need to enable Internet access for nursing records and the ability to use information technology to find valid information.

Leaders perceived that they must ensure the quality of new eHealth services [58]. In the Myllymäki et al. [53] study, telemedicine services were seen as the subject of continuous development from the leaders' perspective, so leaders evaluated telemedicine services regularly. Leaders also brought out the need to participate in creating and using technology in management practices [50]. They pointed out the lack of training in introducing and using equipment and the need for training in acquiring computer systems and equipment [54]. Leaders expressed the wish to learn new technical skills [58] and stressed the lack of skills as one reason for not using certain technologies [59]. Sharpp et al. [2] state that leaders felt that they were expected to be able to use new technologies without any introduction or training, and at the start of positioning as leaders, leaders were frustrated with learning and accessing technology. Leaders were generally confused with the number of mechanisms, websites, and software applications needed to complete their tasks. For example, to compile information related to patient safety, leaders need to access data from multiple technological sources. Additionally, Vandresen et al. [55] pointed out the leaders' ability to use different technologies simultaneously. In their study, leaders also highlighted the lack of training in technology and time management challenges in transforming data into information for decision-making. According to Simpson [60], most leaders cannot advocate for the nursing technology needs in the technology assessment process. Leaders saw that regular sharing of information digitally and in person and handling remote appointments are the primary methods they use in managing telemedicine-related knowledge [53].

Leaders stated that establishing and maintaining relationships was challenging when communicating virtually [2], as is to connect and interact on digital platforms [54], and they need to share information via WhatsApp and communicate in Zoom meetings [50]. Handling a significant amount of emails takes many leaders' time, which has been experienced as a hindrance to effective leadership. Multitasking was essential to keep on top of all the emails and text messages [2]. Leaders pointed out the need for expert advice about the evidence base, available hardware and software, training resources, and implementation strategies of virtual reality [51]. Leaders also stated that mentorship could ease their orientation with new technology [2], and they expressed the need to reach contact and responsible persons [58]. In using data, leaders expressed a lack of support from their superiors [56]. Lack of access to a helpdesk was seen as hindering video interpretation usage [59]. Organisations' strategic guidelines for telemedicine were perceived as a foundation supporting leaders' work [53]. Leaders were poorly informed about legal and ethical issues related to client data, information, and confidentiality [60]. According to Chung et al. [51], leaders perceived the need to develop specific strategies for promoting ethical and safe use of virtual reality. Leaders were unaware of the current evidence base regarding virtual reality [51] and lacked awareness of societal and technological trends, issues, and new developments related to nursing [60].

#### 3.2.2. The Leaders' Need for Digital Competence and Expertise in the Health IT Implementation Process

The second main category consisted of the categories needing more expertise in implementation, motivation, and background in implementation. Leaders stated the need to learn implementation and customer-centered development work and perceived themselves as needing help to plan the implementation of eHealth services [58]. To feel confident implementing virtual reality technology into the nursing practice, leaders recognise gaps in their skills and knowledge to be addressed [51]. According to Bezboruah et al. [57], some leaders implemented health IT in their institution without systematic planning before the implementation process, and in Kujala et al.'s [58] study, leaders perceived that eHealth service implementations were not monitored using measures. Leaders saw that they needed to participate in and support data integration [50] but had time management challenges in implementing technology in nursing management work [55]. Reluctance to change or limited information was the factor referred to when leaders did not implement health IT [57]. Leaders stated that institutional pressures influence health IT adoption, and some leaders said they would only implement additional health IT systems with mandates from the government [57]. Additionally, the process of implementing health IT tended to be imitated by successful counterparts. According to Simpson [60], leaders are knowledgeable in implementing information systems in the nursing practice environment. Still, in the study by Kujala et al. [58], leaders revealed their uncertainty in planning and renewing the eHealth services and operations.

#### 3.2.3. Positive Perceptions towards Technology

The third main category consisted of categories' positive usability experience and positive impact of technology on activities. Leaders pointed out that technology is central to management planning and patient safety interventions [55] and saw IT as a tool for acquiring new knowledge [54]. Leaders reported using data for benchmarking, planning, and decision-making [56]. In the Mottelson et al. [59] study, leaders were generally satisfied with video interpretation and perceived that the technology was easy to use. According to Chung et al. [51], leaders stated that virtual reality technology is comparatively easy to master and perceived as simple. Leaders identified the potential of remote virtual monitoring to be a useful tool [52]. Also, in the Chung et al. [51] study, leaders saw virtual reality as a practical tool and positively accepted new technologies. Myllymäki et al. [53] reported that it is generally essential that leaders possess a positive attitude towards technology and telemedicine and that leaders' willingness to develop and reform telemedicine operations and their sincere interest in their employees promotes the development of telemedicine activities. Sharpp et al. [2] stated that leaders see technology as necessary for leading and cannot perform their tasks without technology. Leaders also pointed out that daily management relies on different technological tools and processes [50] and perceived that utilization and generation of data are necessary for adopting evidence-based practices [56]. Leaders saw that well-organised technology improves patient safety outcomes, saves time and effort, and can help standardise competencies, introduce things, and improve communication [2]. In Vandresen et al. [55] study, leaders perceived that technology facilitates daily processes, communication, data collection, and information sharing, making data more accessible.

#### 3.2.4. Negative Perceptions towards Technology

The fourth main category consisted of categories of negative usability and accessibility experiences and encountering problems related to technological systems. Leaders reported having computer problems in everyday life [54] and having problems related to Internet access [50]. Furthermore, they perceived that the complete use of management technologies in hospitals is hindered by insufficient material resources and a lack of functionality of technological instruments [55]. In the Wong et al. [56] study, leaders expressed that incomplete or inadequately aggregated data challenge the data usability and cause delays in data reporting. They also pointed out that data inaccessibility is a general challenge. Also, in the Vandresen et al. [55] study, leaders saw a lack of interoperability between technological systems as a challenge with using management technologies in hospital settings. Poor system interoperability and data combination were reported as significant drawbacks, according to Wong et al. [56]. In their study, leaders perceived the lack of intraorganizational harmonisation in software usage as a challenge in using data to guide inpatient staffing management decisions. They also saw that data system configurations challenged the timely addressing of events. Inconsistencies in data collection and reporting across different data tools were perceived to hamper effective decision-making in staffing management, as was data fragmentation. In addition, accumulating and compounding data from different data tools were experienced as time-consuming and challenging.

#### 3.2.5. Ability to Act as an Advocate to Implement Technology into Practice

The fifth and final main category consisted of categories training and guidance skills and showing support and example. In Vandresen et al.'s [55] study, leaders saw employees' lack of technology skills and knowledge as a challenge. Kujala et al. [58] study revealed that leaders are concerned about training, engaging, and committing employees to new eHealth services. In the Hawksworth et al. [52] study, leaders saw providing education and advocating remote visual monitoring as a way to enhance its acceptance among professionals, and providing education about remote visual monitoring and advocating it to the professionals were also seen as patient safety strategies. Leaders brought out the need to guide professionals and patients in eHealth and felt that guiding different patient groups in eHealth is challenging [58]. Leaders also expressed fear of employees' resistance to change and negative attitudes regarding eHealth [58]. They felt they needed to consider employees' negative attitudes in the implementation process [51]. The leader's role includes explaining technology's effects on employees' workflow [52], and leaders often use technology to act as an example for employees [53]. Advocating health IT systems' effective applications and functionalities to employees was hindered by the leader's lack of complete information on health IT systems [57]. Leaders' characteristics were seen as important in supporting employees in telemedicine, and good communication between leaders and employees was a key position in successful telemedicine actions [53]. Leaders' support and interest in telemedicine were seen as important for practice, and leaders' roles in telemedicine included supporting and encouraging employees, being positive, and acting as an example [53].

### 3.3. Healthcare Leaders' Digital Competence and Factors Associated with It Based on the Quantitative Data Synthesis

The selected quantitative studies examined the following areas of digital competence: “Use of information communication technology and computer skills [43],” “Knowledge of artificial intelligence and robot nurses [44],” “Attitudes about technology use [48],” “Informatics competencies [47],” “Technological competency as caring in nursing [45],” “Information system support for performance management [46],” and “ICT use [49].”

**Figure 2:**
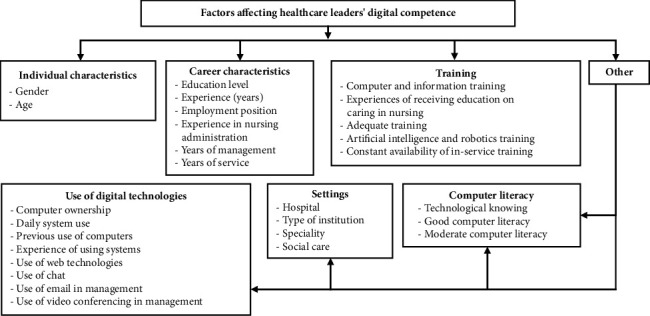
Factors affecting healthcare leaders' digital competence.

Data from the selected quantitative studies present that factors associated with the digital competencies of healthcare leaders include individual characteristics entailing the gender and age group, career characteristics entailing the education level, years of experience, employment position, experience in nursing administration, years of management and years of service and training entailing computer training before current status, information education/training, the experience of receiving education on caring in nursing, artificial intelligence and robot nurse courses, the experience that in-service training is constantly available, and the experience of having adequate in-service training to use information systems. Other factors associated with the digital competencies of healthcare leaders included, for example, technological knowledge, computer and software use and ownership, type of institution and speciality, user experience, and computer literacy (Table 3 and Figure 2).

From individual characteristics, gender and age are associated with the use of information communication technology and computer skills [43], knowledge of artificial intelligence and robot nursing [44], and attitudes about technology use [48]. It was evident that females' computer knowledge was lower than that of males, and the proportion of females not knowing how to use computerised patient monitoring systems was higher than that of males. Therefore, males' ICT ability was higher than females [43]. It is also evident that nurse leaders' age is significantly associated with the use of ICT; nurse leaders aged between 29 and 39 years were able to use ICT in nursing, while nurse leaders aged 50 years or older abilities were found lower, younger nurse leaders were more able to type using keyboards, use software applications for planning and making decisions, compose and send emails, use the Internet to locate and download items, and conduct other computer tasks compared to their older counterparts [43]. Ergin et al. [44] study presented a significant association between participants' gender and the belief that AI and robot nurses would be beneficial for nursing, and it was evident that females believed that AI and robot nurses would be helpful for nursing. The study also found that older nurse leaders (aged 31–40) reported statistically significantly having heard more of the concepts of AI and robot nurses than younger nurse leaders [44]. Also, Martins et al. [48] reported that gender is significantly associated with the perception of utility and ease of use of different ICTs.

From career characteristics, education level is associated significantly with the use of information communication technology and computer skills [43], with informatics competencies [47], and with knowledge of artificial intelligence and robot nurses [44]. In the Adatara et al. [43] study, there was a significant association between nurse leaders' educational level and ICT use, and it was evident that nurse leaders with a bachelor's degree or higher education were using ICT in nursing care more than nurse leaders with lower education. In the Yang et al. [47] study, nurse leaders' education level significantly impacted their informatics competencies, which implies that a higher education level increases nurse leaders' informatics competency. Ergin et al. [44] found a statistically significant difference between the thought that AI and robot nurses would benefit the nursing profession and their educational level, presenting that nurse leaders with undergraduate degrees believed that AI and robot nurses would benefit nursing.

Experience (years/length) and the employment position were significantly associated with the use of information communication technology and computer skills [43], knowledge of artificial intelligence and robot nurses [44], and technological competency as caring in nursing [45]. Higher work experience in years was found to be a decreasing factor in ICT use among nurse leaders, and employment position has a significant association with leaders' computer knowledge, presenting that nurse leaders with higher positions have less computer knowledge compared to those with lower rank [43]. Ergin et al. [44] study presented a significant association between participants' seniority in the position and the employment position and the belief that AI and robot nurses would benefit nursing. It was evident that nurse leaders with 0–15 years of seniority in the position and position as ward supervisors believed that AI and robot nurses would benefit nursing. Additionally, the study presented a significant relationship between the idea that AI and robot nurses would replace nurses and nurse leaders' employment positions; ward supervisors did not think that robots would replace nurses [44]. Nakano et al. [45] reported a significant association between nurse leaders and nurses' technological competency in caring in nursing (TCCN). Their years of experience, presenting that a length of experience of five to less than ten years showed significantly lower TCCNI-R (Technological Competency as Caring in Nursing Instrument-Revised) scores than those with years of experience of 20 to less than 30 years.

Experience in nursing administration associated with informatics competencies [47]. This study showed that nursing administration experience negatively affects nurse leaders' informatics competencies, presenting that having more administrative experience is associated with lower informatics competencies. Years of management and service are associated with attitudes about technology use [48]. Martins et al. [48] found significance in the perceived utility of the discussion groups, the time of experience in the services, and the usefulness of SINAI (a device to access information from other information devices) technology and time of experience in management. The results revealed that leaders with less time of service did not perceive the discussion groups as a useful tool, and this device was not considered useful among most leaders. Additionally, SINAI was also a technology not perceived as useful by leaders with less experience [48].

From training characteristics, computer training before the current position is associated with the use of information communication technology and computer skills [43], information education/training with informatics competencies [47], experiences of receiving education on caring in nursing with technological competency as caring in nursing [45], and taking courses about artificial intelligence and robot nurses with knowledge of artificial intelligence and robot nurses [44]. According to Adatara et al. [43], nurse leaders who had had computer training prior to positioning as a nurse leader possessed more knowledge of ICT use compared to nurse leaders who had not had computer training prior to the position and computer ownership and the use of computers before positioning as a nurse leader were factors influencing the nurse leaders usage of ICT. Yang et al. [47] study presented that nurse leaders' computer and informatics skills were lower than their informatics knowledge. In their study, it was evident that information education or training has a significant impact on nurse leaders' overall informatics competencies. In the experience of receiving education on caring in nursing, about half of the nurse leaders had partaken in an in-service educational activity on Technological Competency as Caring in Nursing (TCCN), and it was evident that nurse leaders were more aware of the TCCN than the staff nurses [45]. In the Ergin et al. [44] study, there was statistical significance between the seniority and educational background and the status of taking courses related to AI and robot nurses, 0–15 years of seniority, and undergraduate degree. Saranto et al. [46] reported that the experience of receiving enough in-service training to use information systems was associated with assessing nursing performance and providing timely evaluation and reporting. Moreover, they reported that the experience of constant availability of in-service training is significantly associated with assessing nursing performance and managing resources.

From other factors associated with the digital competencies of healthcare leaders, hospital, type of institution, and speciality are related to attitudes about technology use [48], technological knowing associated with technological competency as caring in nursing [45], and computer ownership and previous use of computers before appointment as a unit leader associated with the use of information communication technology and computer skills [43]. In the perception of utilities and practicalities of different IT surveyed in the Martins et al. [48] study, the hospital type and setting were seen as significant factors, and it was evident that nurse leaders working in private hospitals perceived greater utility and ease of use of different ICTs, especially regarding e-mail, SINAI, and SISQUAL. For the perception of ease of use of e-mail, intranet, forum, Sape, Sonho, and Aida, and the perception of the utility of e-mail, discussion group, Sape, and Hepic having expertise was proven statistically important [48]. In the Nakano et al. [45] study, results from factor technological knowing showed that nurse leaders recognise the need-to-know patient needs through technology and provide care to the ever-changing patient condition more fully than the staff nurses. In the Adatara et al. [43] study, computer ownership and the use of computers before positioning as a nurse leader influenced the nurse leaders' usage of information and communication technology.

In the Saranto et al. [46] study, good computer literacy and working in social care correlated positively with the three performance management information system features: assessing nursing performance and clinical procedures, managing resources, and providing timely evaluation and reporting. The statement “I use some systems facilitating follow-up of activity every day” is strongly associated with assessing nursing performance and managing resources, as 3 to 6 years of experience of using systems was negatively associated with managing resources. The study by Vaz and Landeiro [49] compared various technologies between the two groups of leaders from different settings (hospital center and nurse leader association). The use of web technologies, chat, e-mail, and video conferencing for any purpose, and management had statistical differences between the groups.

## 4. Discussion

This mixed-methods systematic review aimed to gather the most recent evidence on healthcare leaders' perceptions and experiences of digital competence and associated factors based on qualitative, quantitative, and mixed-methods studies. This review's results present that healthcare leaders' digital competence is a broad concept that includes and is associated by various factors. The original studies included in this review revealed that there is a clear need to develop healthcare knowledge and skills related to digitalisation, to improve their competence regarding digitalisation-related implementation competence, and to raise their level of education since it has been found to have an impact on their use of technology in healthcare settings.

According to the results of the qualitative analysis, there is a need to develop leader's own and others' competence in the digitalisation of healthcare since leaders expressed their knowledge and information-related needs in eHealth and health IT [2, 51–53, 57–60]. Leaders called for information about eHealth services [58], health IT [57], the ability to evaluate digital service activities [53, 58], the competence to communicate through technology [2, 53], and training about technology [2, 58–60]. Leaders also perceived that they need legal and ethical competence to acknowledge the regulatory issues in digitalisation [51, 60], and they brought out especially the lack of knowledge of the evidence base of the digital services and solutions [51, 57, 60]. Still, only a few studies included information about leaders' ethical competence in digitalisation, even though the knowledge of ethical concepts is a significant domain in digital competence [32]. Therefore, future research should aim to gain more information about leaders' ethical competence and legal aspects when utilising digital tools.

The results of this study support previous findings relating to leaders' need to receive continuous education. Training in digital health should emphasise competencies that are pertinent to specific groups of healthcare professionals, their roles, seniority levels, and work settings [32]. The enhancement of digital competence among healthcare leaders necessitates collaborative efforts within government, educational institutes, professional organizations, and healthcare organizations. In order to produce a competent and relevant health management workforce, the current training and development directed for healthcare leaders at the organizational level and the review of higher degree teaching curricula to incorporate emerging digital competency requirements for healthcare leaders need to be changed. Reference [11].) Laukka et al. [18] conclude that leaders' education has not yet been modified to meet rapid changes in digitalisation. Therefore, leaders benefit from additional training or even participating actively in digital service development. Also, Laukka et al. [62] report that leaders may gain specific informatics skills and knowledge needed for decision-making regarding digital health services, for example, via education. In the study by Carson et al. [13], leaders' proficiency in using the EHR system was enhanced through training sessions. Strudwick et al. [10] present that healthcare organizations need to see leaders with specific informatics expertise as essential to effective health information technology decision-making, implementation, optimisation, and evaluation. Supporting leaders' digital competence development through continuous education accelerates advancement opportunities and assists in gaining an appropriate competence [5]. On this account, we recommend that the development of healthcare leaders' competence in technology use via various educational activities is necessary. Most importantly, we suggest that organizations should put effort on leaders' intellectual humility, curiosity, courage, and developmental maturity to enhance digital competence preceding concrete educational activities or training and to direct the necessary developmental activities to relevant leaders.

Our results implicate that healthcare organizations should put effort into implementing and utilising mentoring activities and expert advice [2, 51, 53, 58, 59]. The provision of mentoring and peer support also has the potential to enhance leaders' work well-being [63], yet further research on the suitable and effective mentoring methods and contents to improve healthcare leaders' digital competence is needed. Leaders' attendance in training or other methods of digital competence development is challenged by a lack of resources, such as time, expenses, or broader organizational support. Therefore, the organizations and work units should create more forceful structures and possibilities that enable the identification, evaluation, and development of leader's digital competence to encourage the use of digital technologies to their full extent and indicate a broader support system as the leaders themselves are mostly motivated towards the utilization of new technologies according to the results of our study. In a study by Kulju et al. [64], utilising a multimethod approach, supplementary materials, and opportunities for practicing new skills and exchanging experiences or inquiries seems to offer an effective strategy for delivering digital competence education to healthcare professionals. In light of our findings, we recommend that these factors could also be considered in training healthcare leaders to enhance their digital competence. Moreover, AI concepts should be woven into the curriculum of all healthcare leaders' education programs to enhance the AI literacy and skills of leaders interacting with AI-based systems. This integration is crucial for organizations to enhance current working flow systems, navigate change, and effectively cultivate a culture that evolves with rapid technological advancements [24, 28, 65]. Yet, future research should focus on identifying the specific methods and their effectiveness on leaders' digital competence development, as there is a research gap in this area.

The analyzed qualitative research revealed that healthcare leaders need expertise in the implementation process regarding digitalisation in healthcare settings [51, 57, 58, 60] since leaders brought out the lack of implementation related skills [58], knowledge [51, 57, 58], and motivation [57] needed to implement the health IT into practice. The leaders' lack of motivation to increase the rate of use of health IT has also been recognised in other studies [66]. A review by Ingebrigtsen et al. [20] showed that leaders' IT knowledge positively affected information technology adoption. Their review presented that leaders possessing technical informatics skills and previous experience in IT project management are likely to develop a vision that creates long-standing engagement in the use of IT, are motivated in IT adoption, and are prepared for the adversities regarding implementation. Additionally, previous studies indicate that leaders' full engagement in digital health improves service implementation [62]. However, leaders need more support in role identification and do not understand the implementation procedure [67]. Senior leadership support is crucial for fostering a shared vision among various stakeholders and achieving the desired impact, for example, when implementing AI into healthcare [68]. As healthcare employees' technology acceptance, and thus indirectly their motivation to commit to the current work responsibilities, is reliant on the leader's digital competence [35], it is valuable to find relevant possibilities to improve healthcare leaders' skills, knowledge, and motivation when implementing digital health according to the findings of this review.

The qualitative synthesis revealed additionally that healthcare leaders need to be able to act as advocates to implement technology into practice [51–53, 57, 58]. According to the results, this calls for skills in training and guidance [51, 52, 58] and showing support and example to the employees by advocating for technology [52, 53, 57]. The importance of leaders' actions showing a good example to employees by using technologies in practice has also been evident in other studies [16, 18, 69] and sometimes providing training to the employees by acting as a teacher themselves [62]. Also, Kujala et al. [17] pointed out the frontline leaders' role in sharing the vision with the staff, involving them in the planning, and supporting their positive attitudes in eHealth services implementation. Our results also revealed the healthcare leaders' need to consider professionals' negative attitudes during implementation processes [51, 58], which is similar to the results by Laukka et al. [18]. As leaders' role in supporting digital technology implementation and employee's digital competence are inevitable, we suggest that leaders focus on supporting healthcare professionals through proactive mentoring to ensure a digitally competence workforce and reinforce role clarity [70].

In our review's qualitative synthesis, leaders expressed mostly positive perceptions towards technology with experiencing positive usability of the technology [51–53, 59] and recognising its positive impact [2]. However, according to Sharpp et al. [2], leaders were often disconcerted by the variety of technologies to complete their tasks. They felt that they were expected to know how to use different technologies without introduction or training, which brought out feelings of frustration with accessing and learning technology, especially when starting in a leadership position. Laukka et al. [18] found similar results, that leaders sometimes feel digitalisation is stressful but also an easing element in their work. Moreover, in our study, leaders expressed negative perceptions concerning lack of IT capabilities and physical resources [50, 54, 55], data availability problems [56], poor interoperability of systems [55, 56], and challenges in data combining [56]. Issues with data interoperability and availability and lack of digital competence can induce several risks, such as endangering patient safety, delaying care processes, and challenging timely decision-making as part of managerial responsibilities. Therefore, we propose that leaders must be mindful of the various effects of digitalisation as part of their digital competence.

The analyzed quantitative data presented that leaders' age was significantly associated with their digital competence, implying that younger leaders could use ICT in nursing more than their older counterparts [43]. Moreover, leaders' educational level significantly impacted several researched digital competencies, implicating that higher education, more specifically, a master's degree, promotes leaders' informatics competencies [47]. Higher education positively impacts the use of information communication technology and computer skills [43], which supports our proposal for raising the healthcare leaders' educational level to increase their digital competence. Higher work experience in years was a decreasing factor in ICT use among nurse leaders [43]. More administrative experience was associated with a lower level of informatics competencies [47]. We suggest that there is a need to pay attention to developing the more experienced and older leaders' digital competence, for example, through reverse mentoring, since according to previous research, reverse mentoring is considered an effective approach in developing the senior employees' up-to-datedness with technology [71, 72]. This can potentially support the leaders´ work well-being, satisfaction, and effective functioning in digitalising healthcare in their remaining years of practice yet factors such as authoritative leadership styles in different cultures may challenge the implementation of reverse-mentoring.

The quantitative data also revealed the impact of gender on the digital competence of healthcare leaders, as the results show that females' digital competencies, more specifically in computer and patient monitoring system knowledge, are generally lower than males' [43]. On the other hand, results from Ergin et al. [44] show that females are more optimistic than males about the impact of AI and robot nurses on nursing care. Moreover, one study concluded that female leaders are more heavily influenced by the utility and ease of use of different ICTs [48]. In light of these findings, we recommend paying particular attention to developing female leaders' technological know-how yet means to change the attitude environment relating to the use of new digital solutions should be targeted more vigorously towards male leaders. Still, more research on the effect of the leader's gender on digital competence on a wider scale is needed.

## 5. Limitations and Strengths

Our review has some limitations. In our review, we found only one study referring to the digital competence of nurse managers in social care [46]. Therefore, our results refer only to the digital competencies of healthcare leaders. Some of the relevant studies regarding our inclusion criteria may have been left out of this review even with the systematic process to prevent publication risk and even though we searched four databases and checked the reference lists of the included studies since we left out the grey literature and limited the timeframe and language to English or Finnish. In this review, conducting a meta-analysis was impossible; however, tabulation and data synthesis supported the reliability of the presented data of the chosen studies. Each chosen study's quality assessment uses the JBI quality appraisal tool. One study [59] scored lower than 50% in quality assessment but was still included in the final data synthesis. The sensitivity analysis by excluding that one study was not possible according to the chosen data synthesis methodology. This review summarised the results of studies conducted in different countries. There may be differences in the digitalisation of social care and healthcare between these countries, which need to be recognised in results' generalizability. The PRISMA 2020 statement: a guideline for reporting systematic reviews was used to increase the transparency of this report [73].

## 6. Conclusions

The results of this review indicate that healthcare leaders must possess a wide range of digital competencies to work effectively in the constantly developing healthcare environments. Considering the geographical diversity of the articles incorporated in our review, we can infer that the digital competence of healthcare leaders holds worldwide significance in healthcare management. The research discussed in this review suggests that developing and supporting leaders' digital competencies should be considered in healthcare organizations, research, and education to meet the demands of increasingly digitalising healthcare development work. We see leaders' mainly positive attitudes towards digital competence as a favourable factor for their competence development. So, we are suggesting that leaders should embrace an active role and take responsibility in actively seeking opportunities where their digital competencies can be improved. Simultaneously, leaders should be mindful of the pitfalls of digitalisation's effects and challenges and reflect these aspects as part of their digital competence. We suggest that to increase healthcare leaders' digital competence, healthcare organizations should prioritise the development of policies, structures, and processes to ensure effective design, planning, and implementation of innovative adoption while also dedicating financial resources to support management workforce development.

We also propose that healthcare organizations should develop different models and practices to reinforce and transfer leaders' digital competencies through various activities. In-house training should focus on the specific digital systems to be implemented in the organization before their roll-out, and continuously available user support should be provided. The training should aim to increase healthcare leaders' digital skills needed in their daily work and a comprehensive understanding of the benefits and disadvantages of digital tools in supporting patient-centered, effective, and evidence-based care in digitalised healthcare. Prior to education, leaders' developmental maturity should be investigated to avoid potential waste or inefficient use of resources. We propose that data interoperability and accessibility issues be identified and resolved at the organizational level to help healthcare leaders work in digital environments. As our results showed, leaders' education level significantly impacts their digital competencies, and digital competence should be considered in the curriculum of healthcare leaders. With the increasing use of AI in healthcare, we see that the AI perspective needs to be taken into account as part of healthcare leaders' digital competence, both at the educational and organizational level. In terms of further research, more information is needed on leaders' ethical and legal competencies as part of digital competence and the complexities of introducing AI-based systems to healthcare practice. We also recommend future research to investigate interventions that can support digital competence development of more mature leaders effectively and comprehensively since their digital competencies were found to be lower than those of their younger counterparts.

## Figures and Tables

**Table 1:** Extraction data.

Author(s), year, and country	Purpose	Participants	Methodology (design, data collection, data analysis)	Exposure of interest and outcome (PEO)	Key findings
Reference [47], China	To examine current informatics competency levels of nurse managers and to identify the variables that influence these competencies	Nurse managers in a general hospital affiliated with Harbin Medical University (*n* = 68)	A cross-sectional study A questionnaire. (First part queries about the general characteristics of the respondents, second part investigates nurse managers' informatics competencies using 49 informatics competency items identified by Hart in three categories: computer skills (25 items), informatics knowledge (20 items), and informatics skills (4 items)) Statistical analysis	Levels and variables Informatics competence	The study presents that nurse managers' informatics competency is at a medium level, and the level of their informatics knowledge was significantly higher than their informatics and computer skills. The variability in informatics competencies was determined at 71.2% with information education/training, nursing administration experience, and the level of education, and these were significant factors in affecting nurse managers' informatics competency levels. Higher education and informatics education/training were positively affecting factors, and longer nursing administration experience was shown to negatively affect informatics competency levels

Reference [59], Denmark	To investigate the attitudes to and experiences with video interpretation among charge nurses in a Danish University Hospital	Charge nurses (Position as managers closely involved in determining nursing practice.) (*n* = 99)	Mixed methods An electronic questionnaire (pretested, developed for the study) A descriptive statistics and thematic text condensation	Attitudes and experiences Video interpretation	Most charge nurses using video interpretation expressed satisfaction with the technology and ease of use, and frequent users were more satisfied with video interpretation than rare users. Charge nurses in outpatient clinics were more frequent users compared to the inpatient departments and acute areas. Video interpretation was seen as a tool helping increase the quality of care and communication when there are language barriers, and dialogue and the relationship between the nurses and the patients seemed to be improved by the technology. Some of the charge nurses mentioned that video interpretation helped save time in consultations, but some found the technology complicated to use. Four different themes occurred when asked reasons not to use video interpretation: professional concern, administrative barriers, patient's state of health, and using alternative approaches. Challenges in using video interpretation were mostly related to practical issues: poor Internet connection, technical problems, and lack of equipment

Reference [43], Ghana	To examine the factors that influence the use of information and communication technology among nurse managers	Nursing unit managers (*n* = 108) of the selected health facilities in the Volta region of Ghana who were performing the role of frontline managers	A cross-sectional study design A self-administered questionnaire (developed for the study) Statistical analysis	Factors influencing information and communication technology knowledge and use (sex, age group, years of experience, the rank of the respondents, computer training, computer ownership, and the use of computers before the current position) The use of information and communication technology	Younger nurse managers were significantly more able to use ICT in nursing care than their older counterparts. Bachelor's or higher degree showed increased use of ICT in nursing care. Higher work experience years had a decreased effect on the nurse managers' use of ICT, compared with those with fewer years of work experience. Lower work experience in years showed increased likeliness to use ICT in the workplace, than more work experience in years. Higher knowledge of ICT use was shown to be associated with computer training before the current position

Reference [17], Finland	To examine the current state of eHealth competencies of clinical leaders in two public healthcare organizations in Finland	Clinical leaders (nurse leaders 32%, social worker leaders 25%, physician and dentist leaders 11%, other leaders 13%, and managers 19% (*n* = 98)	Mixed methods An online questionnaire developed for the study with multiple-choice and open-ended questions and a 5-point Likert scale Descriptive statistics for quantitative data, content analysis for responses to the open questions	State eHealth competencies	Leaders need to organize workflows, support healthcare professionals in the change, and make sure that eHealth service quality is good enough and that patients are informed and guided. Leaders' self-perceived eHealth competencies were the strongest regarding security and privacy protection, on which they had recently had training. Most leaders were confident in using eHealth applications and services and leading change and supporting their subordinates' competence. Leaders felt the least confident in communicating about the new eHealth services to patients, customer-centered service development, and planning new eHealth services implementation. Many leaders did not feel confident in their tasks, which indicates the need for training and support; leaders called for more information about eHealth services and their benefits and possibilities. The study indicates the need for training in managing change and planning the implementation of new eHealth services. Leaders have a critical role in ensuring the successful adoption of new eHealth services and supporting healthcare professionals in order to avoid resistance to change

Reference [48], Portugal	To analyze the association between the characteristics of nurse managers and the use of information technologies in Portuguese hospitals	Nurse managers (*n* = 138)	A cross-sectional study Questionnaire Likert scale (developed for the study) Statistical analysis	Associations Use of information technologies	The study presents it is evidence that the perception of ease of use and utility of the nurse managers towards IT is influenced by the scenario in which they work and their socio-occupational characteristics, especially gender, complementary training, and time of experience in services and management. It was also presented that this perception is unique to each IT

Reference [45], Japan	To determine managers' and staff nurses' perceptions regarding the technological competency as caring in nursing (TCCN) theory in general hospitals in Japan	Nurse managers (*n* = 96) and staff nurses (*n* = 325)	A cross-sectional study design A web-based questionnaire using the TCCNI-R, a 5-point Likert scale Statistical analysis	Perceptions Technological competency	The study presents that the nurse managers were more aware of TCCN than the registered nurses. In the factor “technology and caring,” the study presents that nurse managers were more aware of providing care using technology and also about technological competency as an expression of caring in nursing than registered nurses. In the factor of “technological knowing,” nurse managers recognised more fully the need-to-know patient needs through technology than registered nurses

Reference [44], Turkey	To identify nurse managers' opinions on artificial intelligence (AI) and robot nurses	Nurse managers (*n* = 326)	A cross-sectional descriptive study 21-Item online questionnaire form (developed for the study) Statistical analysis	Opinions Artificial intelligence and robot nurses	Most of the nurse managers were aware of the concept of AI and robot nurses and saw that AI would be beneficial for nurses and would reduce nurses' workload, and more than half stated that basic education on AI and robot nurses should be included in university programmes. Half of the nurse managers brought out that robot nurses should only fulfil nurses' orders, and not to be used in providing patient care independently. In case, problems arise in the patient care provided by the robots, the majority of participants stated that engineers, hospitals, and lastly the nurses should take responsibility for the actions of robot nurses; to engineers, the responsibility and safety, and to nurses the ethical responsibility for patient safety and nursing care regarding to robots

Reference [46], Finland	To determine nurse managers' opinions of information system support for performance management	Nurse managers (*n* = 419)	A cross-sectional study design Electronic questionnaire, a 5-point Likert scale Statistical analysis	Knowledge and opinions Information system support for performance management	Good computer literacy and working in social care were positively associated with the features or performance management information systems (assessing nursing performance and clinical procedure, managing resources, and providing timely evaluation and reporting). The use of the systems to facilitate follow-up was strongly associated with assessing nursing performance and managing resources Available in-service training was found to have a positive impact on the leaders' use of the information system

Reference [49], Portugal	To identify the technological profile of nurse managers	Nurse managers (*n* = 74) from two settings: hospital center in the Portuguese north region (*n* = 30) and members of the Portuguese association of nurse managers and leadership (*n* = 44)	An exploratory, descriptive, quantitative study A self-administered questionnaire Statistical analysis	ICT use The management process	Statistically significant differences were found between the groups in the use of web technologies, chat, e-mail, and videoconferencing. Nurse managers from the hospital center had more experience in ICT use, either in general or as part of the management process (e-mail and videoconferencing)

Author(s), year and country	Purpose	Participants	Methodology (design, data collection, and data analysis)	Phenomenon of interest (PiCO)	Key findings

Reference [60], United States	To identify and validate the gaps existing between selected chief nurse executives (CNEs) self-ascribed lived experience information technology competencies and those laid out by the American Organization of Nurse Executives (AONE)	CNE members of the Health Management Academy (HMA) (*n* = 7)	A qualitative study design Ethnographic interviews Thematic content analysis	Gaps Information technology competencies	In the study, five dominant and often interwoven themes emerged: technology knowledge, collaboration, health information technology (HIT) selection, executive leadership, and standardization. CNEs have chosen to bypass, amassing deep technology knowledge, and to look for nurse informaticians and clinical nurse specialists to provide the deep technology knowledge they do not possess. Limited technology knowledge leaves CNEs unable to champion the collection, analysis, and trending of nursing data in a chief medical officer-dominated HIT discussion. CNEs saw that their lack of deep technological knowledge often hindered effective cooperation across cross-departmental and cross-operational lines. CNEs did not see HIT as a strategic decision support tool for their own use but as a tool for nurses in their daily work and as a dashboard for management. CNEs demonstrated competency in the HIT decision-making process related to the evaluation, selection, deployment, and utilization, but the majority of the CNEs did not demonstrate competency to demonstrate an awareness of societal and technological trends, issues, and new developments as they relate to nursing

Reference [57], United States	To explore the attitudes of nursing home administrators and key managerial staff towards health information technology on the process of health IT adoption	Nursing home directors of nursing, case managers, nursing staff, kitchen staff, and administrative interns (*n* = 42) Primary sources of qualitative data are the administrators of the nursing homes	A qualitative case-study methodology Semistructured interviews based on the literature on health IT implementation in health care facilities and observer notes Inductive thematic analysis by integrating TAM and UTAUT model	Attitudes of nursing home administrators towards health information technology	The actions of the other nursing homes were seen as highly influencing social factors in managerial decision-making in implementing health IT. The administrator's conception of the potential benefits and usefulness of health IT was shown to support the procurement of technology adoption. Some nurse managers reported that without mandates from governments to adopt health IT, they have no intentions of health IT adoption and will have no systematic exploration of health IT implementations' positive effects on the quality of care and efficiency. Administrators' health IT implementation attitudes were affected by the price of the health IT system and the following upgrades on the computers and the staff training costs, the perception of staff's resistance to change, lack of strategic planning, evaluation, and cost-benefit analysis, the absence of regulatory pressures and satisfaction with current processes before the health IT. Administrators in nursing homes without health IT adoption had apathetic attitudes towards the change and innovation

Reference [2], United States	To explore how nurse managers use ICT to lead and communicate virtually in U.S. healthcare systems	Nurse managers of nursing service surgery line departments within a large, national healthcare system (*n* = 16)	A descriptive, exploratory qualitative design In-person, face-to-face interviews, a focus group, and survey questions on background information Statistical and inductive thematic descriptive analysis	Nurse managers' use of ICT in virtual leading and communication	Nurse managers are dependent on technology to complete all aspects of their tasks and communicate and organize. Nurse managers were often overwhelmed by the amount of emails, messages, software applications, websites, mechanisms, and devices needed to complete their tasks. Nurse managers called for personal mentorship and practical education on how to master technology use in their work effectively; they need to understand how to use the technology and the data it provides and to know how to apply the data for meaningful difference in organization and to impact patient care

Reference [51], Australia	To explore the perspectives of clinicians and service managers working in private mental healthcare regarding virtual reality (VR) use, including potential implementation barriers and facilitators	Clinicians (*n* = 14) and service managers (*n* = 5), aged 28–70 years working in a major private mental health hospital in Victoria, Australia	A qualitative study design Semistructured qualitative interviews Inductive thematic content analysis	The perspectives of staff in the use of therapeutic VR	To feel confident implementing VR technology, clinicians and managers identified knowledge and skills gaps; clinicians needed training in technical VR skills, assessing patient suitability, and managing ethical and safety risks, and managers needed “expert advice” to be informed about the evidence base, available hardware and software, training resources, and implementation strategies. Generally, clinicians and managers showed positive attitudes towards embracing new technologies. Staff perceived VR to be a relatively simple technology that clinicians and patients could easily learn to use, and some felt it makes clinical work easier. A service culture that values patient-centered care and innovation positively affected the staff's overall positive attitudes to therapeutic VR. Perceived usability issues of VR systems were seen as barriers to their implementation

Reference [52], United States	To better understand direct care nurses and nurse leaders' perceptions of the barriers and facilitators that influence nurses' acceptance and use of remote visual monitoring (RVM) technology	Nurse leaders (*n* = 6) and direct care nurses (*n* = 7)	A qualitative descriptive study design Four semistructured focus group interviews (two nurse leader groups and two direct care nurse groups) Conventional content analysis	Perceptions of the factors related to nurses' acceptance and use of RVM technology	The content analysis resulted in five themes that addressed the barriers and facilitators to nurses' acceptance and use of RVM technology: (1) contextual human factors that impact nurse acceptance; (2) facilitators and barriers related to RVM's functionality; (3) nurse leaders' role in maintaining device availability and efficient use; (4) nurse leaders' role in promoting adoption of the technology; and (5) nurse leaders' role in valuing nursing professional judgement. Many leaders described advocating for RVM and providing education about the RVM protocol and its functions as ways to enhance its acceptance among nurses

Reference [53], Finland	To describe competence management in telemedicine from the perspective of health and social care frontline leaders	Frontline leaders from primary health care, specialised medical care, and social care (*n* = 10)	A qualitative study design Thematic interviews Inductive content analysis	Competence management in telemedicine	The interest and support of senior management were seen as important in telemedicine, and the role of frontline leaders is to support, encourage, and be positive and an example for the staff providing telemedicine. Leaders' positive attitude regarding technology and telemedicine was evident to be generally important, and leaders often run dedicated technology and telemedicine to give an example to professionals. When supporting the telemedicine provided by the professionals, leaders' characteristics such as credibility and easy accessibility were seen as important. Leaders perceived assessing professionals' competence in telemedicine to be challenging, but they recognised the different skills required for professionals in telemedicine. Good interaction between the leader and the professionals was seen as a key to successful telemedicine and competence management

Reference [55], Brazil and Portugal	To describe the potentialities and difficulties mentioned by nurse managers in the use of technologies in hospitals	Nurse managers (*n* = 71)	A descriptive-exploratory qualitative study design Semistructured interviews Thematic content analysis and work process theory	Potentialities and difficulties in the use of technologies	The study pointed out that leaders need to be able to use different technologies simultaneously. Leaders pointed out that the use of technology is central to management planning and patient safety interventions and perceived that technology facilitates daily processes, communication, data collection, and the sharing of information and makes data more accessible. They highlighted the lack of training on technology and the challenges of time management in transforming data into information for decision-making. Leaders had time management challenges in the implementation of technology in nursing management work. They perceived that complete use of management technologies in hospitals is hindered by insufficient material resources and lack of functionality of technological instruments and saw a lack of interoperability between technological systems as a challenge. They also saw employees' lack of technology skills and knowledge as a challenge

Reference [54], Norway and Finland	To map what experiences nurse leaders have encountered in connection with the change work that political decisions and reforms have created within the healthcare sector in the last 25 years	Nurse leaders (*n* = 8) from primary and specialist health services	A qualitative narrative study design Individual interviews with an open interview guide A four-step text condensation analysis	Experiences regarding the organizational changes	Leaders pointed out the lack of training in the introduction and use of equipment, as well as the need for training in the acquisition of computer systems and equipment. They perceived connecting and interacting on digital platforms as challenging. Leaders saw IT as a tool for acquiring new knowledge but reported having computer problems in everyday life

Reference [50], Indonesia	To explore the perceived core competencies of Indonesian first-line nurse managers within the context of the postpandemic era	Head nurses (*n* = 7) in a public hospital	A qualitative descriptive study design Face-to-face interviews Thematic analysis	Perceived core competencies	One of the four perceived core competencies was technological core competencies. Leaders perceived technological skills' improvement as a necessity and brought out the need to understand technology and how to use technological equipment, the knowledge how to use technological tools to support their work, and the need to understand online systems to be able to guide patients in their use. They also expressed the need to enable Internet access for nursing records and the ability to use information technology to find valid information. Leaders also brought out the need to participate in the creation and use of technology in management practices and pointed out that daily management relies on different technological tools and processes. Leaders reported having problems related to Internet access

Reference [56], United States	To investigate how nurse leaders experience using data to guide their inpatient staffing management decisions in the veterans' health administration	Veterans health administration nurse leaders (*n* = 27) across five management levels	A qualitative descriptive study design One-on-one, semistructured interviews Constant comparative method	Experiences in data usage for inpatient staffing management decisions	Leaders lacked the knowledge about data availability and perceived a lack of appropriate user education, guidance in the use of data, and support from their superiors. Leaders expressed that incomplete or inadequately aggregated data challenge the data usability and cause delays in data reporting. They also pointed out that data inaccessibility is a general challenge in the process. Poor system interoperability and data combination were reported as significant drawbacks

**Table 2:** Results of the content analysis of healthcare leaders' experiences with digital competence.

Sub-categories (*n *=* *26)	Categories (*n *=* *10)	Main categories (*n *=* *5)
Need for information and knowledge about eHealth services Need for knowledge in health IT Need to be able to evaluate digital service activities Need for training in technology Need to know how to communicate through technology Need for mentoring/expert advice with technology Need for legal and ethical competence Lack of knowledge on an evidence base	Knowledge and information-related needs in eHealth and health IT The need for knowledge of the evidence base and regulatory expertise	The need for developing leader's own, professionals' and patients' competence in the digitalisation of healthcare

Lack of implementation skills Lack of implementation knowledge Lack of motivation to implement health IT Need to know how to plan and renew eHealth services	Lack of expertise in implementation Motivation and background in implementation	The leaders need digital competence and expertise in the health IT implementation process

Technologies are easy to use Technology is a valuable and practical tool Openness towards technology Technology is necessary Technology improves operations	Positive usability experience Positive impact of technology on activities	Positive perceptions towards technology

Lack in IT functionality and material resources Data availability problems Poor interoperability of systems Challenges in data combining	Negative usability and accessibility experiences Encountering problems related to technological systems	Negative perceptions towards technology

Need to have the ability to educate professionals about technology Need for guidance skills in eHealth Need to take account of negative attitudes of professionals during implementation Need to advocate for technology to professionals Need to support professionals with technologies	Training and guidance skills Showing support and example	Ability to act as an advocate to implement technology into practice

**Table 3:** Quantitative data.

Factors	Outcomes								
	Digital competence								
	Use of information communication technology, computer skills (1)	Knowledge of artificial intelligence and robot nurses (2)	Attitudes about technology use (3)	Informatics competencies (4)	Technological competency as caring in nursing (5)	Information system support: assessing performance (6)	Information system support: managing resources (6)	Information system support: evaluation and reporting (6)	ICT use (7)
	*n* = 108	*n* = 326	*n* = 138	*n* = 68	*n* = 96	*n* = 419	“	“	*n*=74
*Individual characteristics*									
Gender (%)	p < 0.0001–0.0010	*p*=0.0010–0.8970	*p* < 0.0001–0.0950						
Female	68.5	83.1	56.5						
Male	31.5	16.9	43.5						
Age group (%)	*p* < 0.0001	*p* < 0.0020–0.5100							
20–39 y	48.1								
40–49 y	30.6								
50–59 y	21.3								
≤30 y		27.6							
≥31 y		72.4							

*Career characteristics*									
Education level (%)	*p* < 0.0001	*p* < 0.0001–0.3570		*p* < 0.0001					
State registered nursing (SRN)	27.8								
Diploma	17.6								
Degree	16.7								
Postgraduate	38.0								
Associate degree		12.3		64.7					
Undergraduate/bachelor's degree		50.3		35.3					
Graduate/master's degree		37.4							
Experience (years) (%)	*p* < 0.0001	*p* < 0.0001–0.1000			*p* < 0.0001				
<5 y	9.3				0.0				
5–10 y	14.8				2.1				
11–20 y	58.4				13.5				
21+ y	17.6				46.9				
30+ y					37.5				
0–15 y		53.7							
≥16 y		46.3							
Employment position (%)	*p* < 0.0001	*p*=0.0001–0.4330					*p* < 0.0500		
Nursing officer	6.5								
Senior nursing officer	38.0								
Principal nursing officer	19.4								
Deputy director of nursing services	8.3								
Healthcare services director		4.9							
Healthcare services deputy director		2.8							
Ward supervisor		92.3							
Assistant head nurse							25.1		
Experience in nursing administration (years)				*p* < 0.0001					
Mean (SD)				7.26 (4.66)					
Years of management			*p*=0.0002						
Mean (min-max)			11.4 (6.9–30)						
Years of service			*p*=0.0040						
Mean (min-max)			8 (7.8–35)						

*Training*									
Computer training before the current position	*p* < 0.0001								
Information education/training				*p*=0.0010					
Experiences of receiving education on caring in nursing					*p*=0.0020				
Taking courses about artificial intelligence and robot nurses		*p* > 0.0001							
Adequate in-service training to use information systems						*p* < 0.0010		*p* < 0.0010	
Constant availability of in-service training						*p* < 0.0500	*p* < 0.0100		

*Other*									
Technological knowing					*p* < 0.0010				
Computer ownership	*p* < 0.0001								
Previous use of computers before appointment as a unit manager	*p* < 0.0001								
Hospital			*p* < 0.0001/0.0040						
Type of institution			*p* < 0.0001/0.0020						
Speciality			*p* < 0.0001/0.0030						
Social care						*p* < 0.0500	*p* < 0.0100	*p* < 0.0100	
Experience of using systems (3–6 yrs)							*p* < 0.0500		
Daily system use to facilitate follow-up						*p* < 0.0010	*p* < 0.0010		
Good computer literacy						*p* < 0.0500	*p* < 0.0500	*p* < 0.0500	
Moderate computer literacy						*p* < 0.0500			
Use of web technologies									*p*=0.0030
Use of chat									*p*=0.0030
Use of e-mail in management									*p*=0.0290
Use of video conferencing in management									*p*=0.0260

1 = [43], 2 = [44], 3 = [48], 4 = [47], 5 = [45], 6 = [46], 7 = [49].

## Data Availability

All data generated in this study are presented in this article.

## Supplementary Materials

Supplementary Materials Supplementary file 1. Search strategies used for all four databases (PubMed, CINAHL, Medic, and Scopus) to retrieve the relevant, original studies. Supplementary file 2. Assessment according to the JBI Critical Appraisal Checklists of the methodological quality of the included studies (*n* = 19).
