# Supplementary material for: Digital Competence among Healthcare Leaders: A Mixed-Methods Systematic Review
Source: J Nurs Manag. 2024 Jul 30;2024:8435248. doi: 10.1155/2024/8435248 (PMC11919023; doi:10.1155/2024/8435248)
Supplement: Supplementary Materials — Supplementary file 1. Search strategies used for all four databases (PubMed, CINAHL, Medic, and Scopus) to retrieve the relevant, original studies. Supplementary file 2. Assessment according to the JBI Critical Appraisal Checklists of the methodological quality of the included studies (n = 19). [file 8435248.f1.zip › Supplementary file 1. Search strategies (1).docx]

Supplementary file 1. Search strategy used in the electronic databases.

| Search strategy | |
| --- | --- |
| *PubMed* | ("Nurse Administrators"[Mesh] OR “front-line leader*” OR “nurse leader*” OR “clinical leader*” OR “nurse manag*” OR "nurse director*" OR “ward manager*” OR “head nurse*” OR “nurse middle manager*" OR "nursing manag*" OR "nurse supervisor*" OR "chief nurs*" OR “nurse administ*” OR “nursing administ*”) AND ("Telemedicine"[Mesh] OR "Information Technology"[Mesh] OR "Medical Informatics"[Mesh] OR "Nursing Informatics"[Mesh] OR "Computing Methodologies"[Mesh] OR informatics OR telemedicine OR telehealth OR digital* OR technolog* OR mobile OR online OR application* OR "information system*" OR "information network*" OR “information technology” OR “information communication technology” OR ICT OR virtual OR internet OR "m-health" OR "e-health" OR "mhealth" OR "ehealth" OR "connected health" OR "web-based" OR comput*) AND ("Professional Competence"[Mesh] OR "Attitude of Health Personnel"[Mesh] OR competenc* OR knowledge OR skill* OR attribute* OR expertise OR knowhow OR capabilit* OR capacit* OR qualification* OR abilit* OR literac* OR attitude* OR motivation OR perception* OR experienc* OR opinion* OR thought* OR feeling* OR belief*) AND ("Health Occupations"[Mesh] OR nurs* OR health OR healthcare OR hospital OR "Health Services Administration"[Mesh])  Records identified (14.10.2022) n = 1991  Additional search (2.1.2024) n = 476 |
| *CINAHL* | ( (MH “Nurse Administrators+”) OR “front-line leader*” OR “nurse leader*” OR “clinical leader*” OR “nurse manag*” OR "nurse director*" OR “ward manager*” OR “head nurse*” OR “nurse middle manager*" OR "nursing manag*" OR "nurse supervisor*" OR "chief nurs*" OR “nurse administ*” OR “nursing administ*”) AND ( (MH "Telehealth+") OR (MH "Information Technology+") OR (MH "Medical Informatics") OR (MH "Nursing Informatics") OR (MH "Computing Methodologies+") OR informatics OR telemedicine OR telehealth OR digital* OR technolog* OR mobile OR online OR application* OR "information system*" OR "information network*" OR “information technology” OR “information communication technology” OR ICT OR virtual OR internet OR "m-health" OR "e-health" OR "mhealth" OR "ehealth" OR "connected health" OR "web-based" OR comput* ) AND ( (MH "Professional Competence+") OR (MH "Attitude of Health Personnel+") OR ( competenc* OR knowledge OR skill* OR attribute* OR expertise OR knowhow OR capabilit* OR capacit* OR qualification* OR abilit* OR literac* OR attitude* OR motivation OR perception* OR experienc* OR opinion* OR thought* OR feeling* OR belief* ) ) AND ( (MH “Health Occupations+”) OR (nurs* OR health OR healthcare OR hospital)) OR (MH “Health Services Administration+”) )  Records identified (14.10.2022) n = 1521  Additional search (2.1.2024) n = 233 |
| *Medic* | (osastonhoi* OR apulaisosastonhoi* OR ylihoitaj* OR esimieh* OR johtaj* OR esihenk* OR “front-line leader*” OR “nurse leader*” OR “clinical leader*” OR “nurse manag*” OR "nurse director*" OR “ward manager*” OR “head nurse*” OR “nurse middle manager*" OR "nursing manag*" OR "nurse supervisor*" OR "chief nurs*" OR “nurse administ*” OR “nursing administ*) AND (digi* OR telelääk* OR telehealth* OR teknolog* OR technolog* OR mobiil* OR mobil* OR online* OR ict OR ”tieto- ja viestintätekniikka*” OR tietotekniik* OR tietokon* OR computer* OR internet* OR www OR ehealth OR e-health OR m-health OR mhealth OR verkkopohjai* OR web-based OR sähköi*) AND (competenc* OR kompetenss*OR kelpoisuu* OR pätevyy* OR kyky* OR kyvy* OR knowledge OR tieto* OR tiedo* OR tietämy*OR kyvykkyy* OR skill* OR taito OR taido* OR attribute* OR ominaisuu* OR expertise OR asiantunt* OR ammattitai* OR knowhow OR capabilit* OR capacit* OR qualification* OR abilit*OR literac* OR attitude* OR asen* OR motivation* OR motivaatio* OR perception* OR käsity* OR näkemy* OR experienc* OR kokem* OR opinion* OR mielipi* OR arvio* OR thought* OR ajatu* OR feeling* OR tunne* OR tunte* OR belief* OR uskom*)  Records identified (14.10.2022) n = 100  Additional search (2.1.2024) n =15 |
| *Scopus* | (TITLE-ABS-KEY (“front-line leader*” OR “nurse leader*” OR “clinical leader*” OR “nurse manag*” OR "nurse director*" OR “ward manager*” OR “head nurse*” OR “nurse middle manager*" OR "nursing manag*" OR "nurse supervisor*" OR "chief nurs*" OR “nurse administ*” OR “nursing administ*”) AND TITLE-ABS-KEY (nurs* OR health OR healthcare OR hospital) AND TITLE-ABS-KEY (informatics OR telemedicine OR telehealth OR digital* OR technolog* OR mobile OR online OR application* OR "information system*" OR "information network*" OR “information technology” OR “information communication technology” OR ICT OR virtual OR internet OR "m-health" OR "e-health" OR "mhealth" OR "ehealth" OR "connected health" OR "web-based" OR comput* ) AND TITLE-ABS-KEY ( competenc* OR knowledge OR skill* OR attribute* OR expertise OR knowhow OR capabilit* OR capacit* OR qualification* OR abilit* OR literac* OR attitude* OR motivation OR perception* OR experienc* OR opinion* OR thought* OR feeling* OR belief* )  Records identified (14.10.2022) n = 1443  Additional search (2.1.2024) n =515 |
