# Supplementary material for: Digital Competence among Healthcare Leaders: A Mixed-Methods Systematic Review
Source: J Nurs Manag. 2024 Jul 30;2024:8435248. doi: 10.1155/2024/8435248 (PMC11919023; doi:10.1155/2024/8435248)
Supplement: Supplementary Materials — Supplementary file 1. Search strategies used for all four databases (PubMed, CINAHL, Medic, and Scopus) to retrieve the relevant, original studies. Supplementary file 2. Assessment according to the JBI Critical Appraisal Checklists of the methodological quality of the included studies (n = 19). [file 8435248.f1.zip › Supplementary file 2. Quality appraisal (1).docx]

Supplementary file 2. Quality appraisal

| JBI Critical Appraisal Checklist for Analytical Cross-Sectional Studies | Adatara et al., 2019 | Ergin et al., 2022 | Kujala et al., 2019 | Martins et al., 2020 | Mottelson et al., 2018 | Nakano et al., 2021 | Saranto et al., 2022 | Vaz & Landeiro, 2022 | Yang et al., 2014 |
| --- | --- | --- | --- | --- | --- | --- | --- | --- | --- |
| Were the criteria for inclusion in the sample clearly defined? | Yes | Yes | Yes | Yes | Unclear | Yes | Yes | Yes | No |
| Were the study subjects and the setting described in detail? | Yes | Yes | Yes | Yes | Yes | Yes | Yes | Yes | Yes |
| Was the exposure measured in a valid and reliable way? | Yes | Yes | Yes | Unclear | Unclear | Yes | Yes | Unclear | Yes |
| Were objective, standard criteria used for measurement of the condition? | Yes | Yes | Yes | Unclear | Unclear | Yes | Yes | Yes | Yes |
| Were confounding factors identified? | No | No | No | No | No | No | Unclear | No | No |
| Were strategies to deal with confounding factors stated? | No | No | No | No | No | No | Unclear | No | No |
| Were the outcomes measured in a valid and reliable way? | Yes | Yes | Yes | Yes | Yes | Yes | Yes | No | Yes |
| Was appropriate statistical analysis used? | Yes | Yes | Yes | Yes | Yes | Yes | Yes | Yes | Yes |
| % | 75% | 75% | 75% | 50% | 37,5% | 75% | 75% | 50% | 62,5% |

| JBI Critical Appraisal Checklist for Qualitative Research | Bezboruah et al., 2014 | Chung et al., 2022 | Frilund et al., 2023 | Gunawan et al., 2023 | Hawksworth et al., 2022 | Myllymäki et al., 2022 | Sharpp et al., 2019 | Simpson, 2013 | Kujala et al., 2019 | Mottelson et al., 2018 | Vandresen et al., 2022 | Wong et al., 2023 |
| --- | --- | --- | --- | --- | --- | --- | --- | --- | --- | --- | --- | --- |
| Is there congruity between the stated philosophical perspective and the research methodology? | Yes | Yes | Yes | Yes | Yes | Yes | Yes | Yes | Yes | Yes | Yes | Yes |
| Is there congruity between the research methodology and the research question or objectives? | Yes | Yes | Yes | Yes | Yes | Yes | Yes | Yes | Yes | Yes | Yes | Yes |
| Is there congruity between the research methodology and the methods used to collect data? | Yes | Yes | Yes | Yes | Yes | Yes | Yes | Yes | Yes | Yes | Yes | Yes |
| Is there congruity between the research methodology and the representation and analysis of data? | Yes | Yes | Yes | Yes | Yes | Yes | Yes | Yes | Yes | Yes | Yes | Yes |
| Is there congruity between the research methodology and the interpretation of results? | Yes | Yes | Yes | Yes | Yes | Yes | Yes | Yes | Yes | Yes | Yes | Yes |
| Is there a statement locating the researcher culturally or theoretically? | No | No | No | Yes | No | No | Unclear | No | No | No | No | Yes |
| Is the influence of the researcher on the research, and vice- versa, addressed? | No | No | No | Yes | No | Unclear | No | No | No | No | No | No |
| Are participants, and their voices, adequately represented? | Yes | Yes | Yes | Yes | Yes | Yes | Yes | Yes | No | Yes | Yes | No |
| Is the research ethical according to current criteria or, for recent studies, and is there evidence of ethical approval by an appropriate body? | Unclear | Yes | Yes | Yes | Yes | Yes | Yes | Unclear | Unclear | Yes | Yes | Yes |
| Do the conclusions drawn in the research report flow from the analysis, or interpretation, of the data? | Yes | Yes | Yes | Yes | Yes | Yes | Yes | Yes | Yes | Yes | Yes | Yes |
| % | 70% | 80% | 80% | 100% | 80% | 80% | 80% | 70% | 60% | 80% | 80% | 80% |
